# Mitigating cellular aging and enhancing cognitive functionality: visual arts-mediated Cognitive Activation Therapy in neurocognitive disorders

**DOI:** 10.3389/fnagi.2024.1354025

**Published:** 2024-03-08

**Authors:** Manuela Campisi, Luana Cannella, Dilek Celik, Carlo Gabelli, Donata Gollin, Marco Simoni, Cristina Ruaro, Elena Fantinato, Sofia Pavanello

**Affiliations:** ^1^Occupational Medicine, Department of Cardio-Thoraco-Vascular Sciences and Public Health, University of Padua, Padua, Italy; ^2^Regional Centre for the Aging Brain (CRIC), University Hospital of Padua, Padua, Italy; ^3^University Hospital of Padua, Padua, Italy

**Keywords:** neurocognitive disorders, dementia, Alzheimer’s disease, biological aging, DNA methylation age, leukocytes telomere length, Cognitive Activation Therapy, visual art

## Abstract

The growing phenomenon of population aging is redefining demographic dynamics, intensifying age-related conditions, especially dementia, projected to triple by 2050 with an enormous global economic burden. This study investigates visual arts-mediated Cognitive Activation Therapy (CAT) as a non-pharmacological CAT intervention targets both biological aging [leukocyte telomere length (LTL), DNA methylation age (DNAmAge)] and cognitive functionality. Aligning with a broader trend of integrating non-pharmacological approaches into dementia care. The longitudinal study involved 20 patients with mild to moderate neurocognitive disorders. Cognitive and functional assessments, and biological aging markers -i.e., LTL and DNAmAge- were analyzed before and after CAT intervention. Change in LTL was positively correlated with days of treatment (*p* =0.0518). LTL significantly elongated after intervention (*p* =0.0269), especially in men (*p* =0.0142), correlating with younger age (*p* =0.0357), and higher education (*p* =0.0008). DNAmAge remained instead stable post-treatment. Cognitive and functional improvements were observed for Copy of complex geometric figure, Progressive Silhouettes, Position Discrimination, Communication Activities of Daily Living—Second edition, Direct Functional Status (*p* < 0.0001) and Object decision (*p* =0.0594), but no correlations were found between LTL and cognitive gains. Visual arts-mediated CAT effectively mitigates cellular aging, especially in men, by elongating LTL. These findings underscore the potential of non-pharmacological interventions in enhancing cognitive and functional status and general well-being in dementia care. Further research with larger and longer-term studies is essential for validation.

## 1 Introduction

Population aging emerges as a pivotal phenomenon of our era, reshaping the demographic landscape. This shift in age distribution is poised to amplify age-related conditions, particularly dementia (World Health Organization, 2023), projected to triple by 2050 according to the WHO (Global Dementia Observatory, 2023). Currently, dementia, ranking as the seventh leading cause of death, exacts substantial physical, psychological, social, and economic tolls on individuals, caregivers, families, and society. The global cost surpassed 1.3 trillion US dollars in 2019 (Word Health Organization, 2022; Global Dementia Observatory, 2023). Dementia, stemming from various diseases inflicting damage to the brain and inducing cognitive decline, intricately intertwines with age-related modifications in cellular energy metabolism (Błaszczyk, 2022). Alzheimer’s disease (AD) constitutes the most prevalent form of dementia, encompassing 60–70% of cases, although other neurocognitive disorders are also encompassed (Word Health Organization, 2022).

Aging, on a biological level, results from the cumulative effects of molecular and cellular damage, marked by specific hallmarks such as telomere length (TL) and DNA methylation age (DNAmAge) (López-Otín et al., 2023), which are also considered the early biomarkers of cellular aging (Jylhävä et al., 2017). Telomeres, acting as mitotic clocks, shorten with each cell division, contributing to cellular senescence or death (Srinivas et al., 2020). Leukocyte TL (LTL) is considered as an estimator of mitotic cellular aging (Campisi et al., 2021). DNAmAge, assessed by methylation levels at a species-specific subset of cytosine–guanine dyads (CpG), correlates strongly with chronological age, representing the basis of the “epigenetic clock” theory (Horvath and Raj, 2018). An increase in DNAmAge is indicative of altered biological functions and increased risk of morbidity and mortality (Fransquet et al., 2019).

Research suggests that LTL may play a role in neurodegeneration and disorders like (AD), the primary age-related neurodegenerative condition (Levstek et al., 2020). Previous studies have shown conflicting LTL-AD associations, potentially due to small sample sizes and the presence of other neurodegenerative conditions in AD patients, as well as the inclusion of possible preclinical AD cases (Forero et al., 2016; Scarabino et al., 2017). A recent meta-analysis has reported a connection between AD and shorter LTL (Fu et al., 2022). Furthermore, longer LTL have linked with improved general cognition (Zhan et al., 2018) and performance in cognitive domains (Hägg et al., 2017; Gampawar et al., 2020). Limited population-based studies have explored LTL’s connection to brain Magnetic resonance imaging (MRI) features (King et al., 2014; Suchy-Dicey et al., 2018; Gampawar et al., 2020), with one recent meta-analysis showing that longer LTL is associated with larger whole brain and hippocampus volumes but not white matter (Gampawar et al., 2022). In contrast, critically short LTL has been associated with a more rapid cognitive decline and an increased likelihood of transitioning from mild cognitive impairment to AD (Koh et al., 2020). Less study on the association between DNAmAge and dementia remains controversial. A systematic review found no correlation between DNAmAge and dementia or AD (Zhou et al., 2022), while, an elevated risk of dementia has recently been associated with advanced biological aging as measured by the Horvath epigenetic clocks (Sugden et al., 2022).

**Figure 1:**
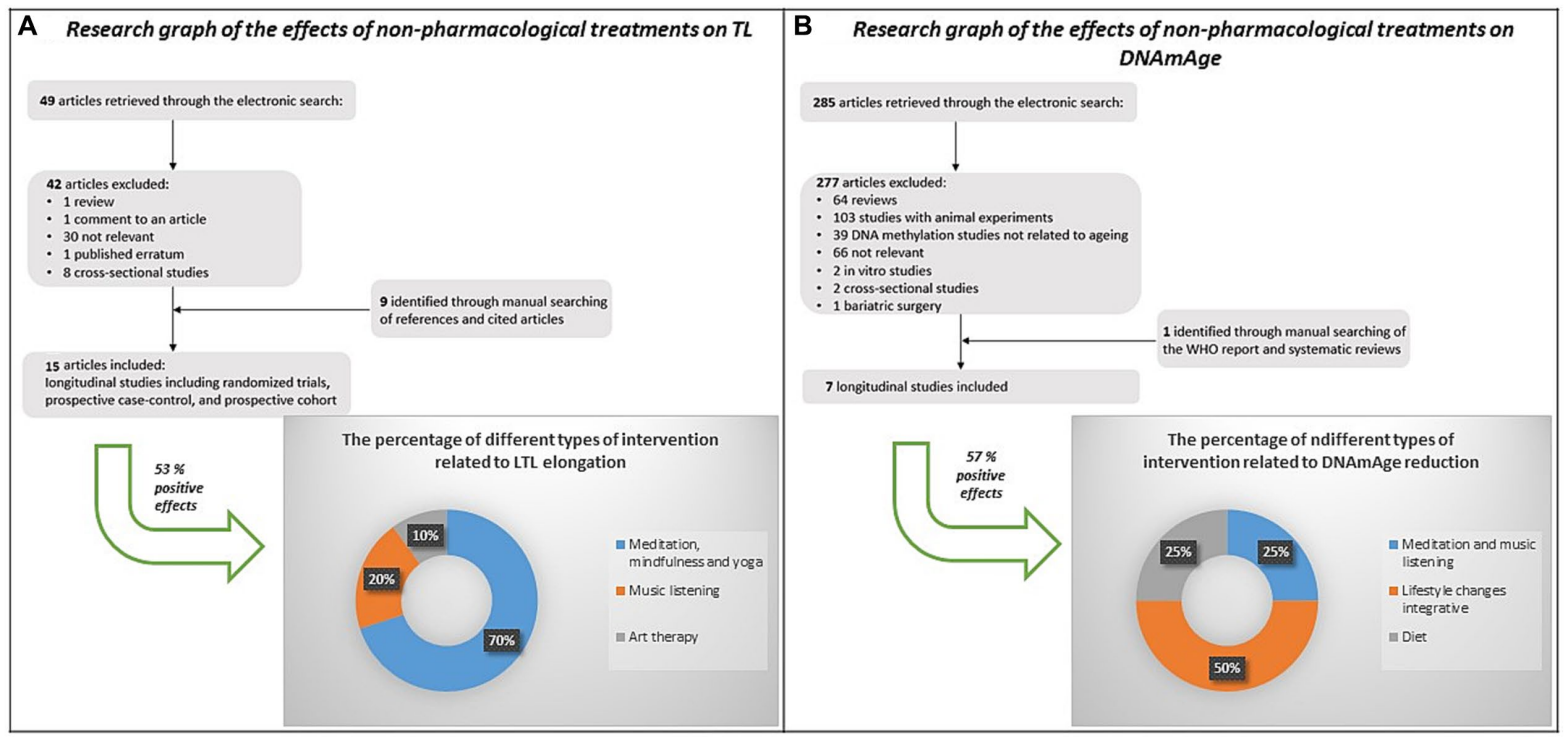
Strategy, selection criteria and results of a literature review on the effects of non-pharmacological interventions on LTL and DNAmAge. Out of the 15 studies found on the effects of non-pharmacological interventions on LTL, 53% (n=8) showed a positive effect, mainly due to mindfulness, meditation and yoga (light blue, 70%), music listening (orange, 20%), and art therapy (light grey, 10%). Out of the 7 studies found on the effects of non-pharmacological interventions on DNAmAge, 57% (n=4) showed a positive effect, mainly due to lifestyle changes integrative (orange, 50%), meditation and music listening (light blue - 25%), and diet (light grey - 25%).

Notably, discoveries even from our laboratory (Pavanello et al., 2019) demonstrate the reversibility of biological aging, suggesting that non-pharmacological interventions can slow down aging. Our literature review (Figure 1) indicated that non-pharmacological treatment effects LTL elongation primarily due to mindfulness, meditation and yoga (Thimmapuram et al., 2017; Conklin et al., 2018; Innes et al., 2018; Tolahunase et al., 2018; Le Nguyen et al., 2019; Pavanello et al., 2019; Athanasopoulou et al., 2021), and music listening (Innes et al., 2018; Pavanello et al., 2019). Only one study considered art therapy (Mahendran et al., 2018). DNAmAge reduction was associated with improved lifestyle changes (Fitzgerald et al., 2021; Yaskolka Meir et al., 2021), diet (Gensous et al., 2020), and meditation and music listening (Pavanello et al., 2019). No study explored the effect of art therapy on DNAmAge.

The objective of this study is to explore the effectiveness of visual arts-mediated Cognitive Activation Therapy (CAT) as a non-pharmacological intervention in individuals with mild to moderate neurocognitive disorders. This intervention aims to counteract both biological aging and the cognitive/functional state of patients with neurocognitive disorders. This research aligns with the growing interest in incorporating non-pharmacological treatments into dementia care to enhance cognitive performance, as stated in the study by Johnson and Wiseheart (Johnson et al., 2020).

## 2 Materials and methods

### 2.1 Study design

A longitudinal study was carried out involving 20 patients, aged between 64 and 85 years, with a neurocognitive disorder in the mild to moderate phase of the disease. All patients were recruited at the Regional Centre for Cerebral Aging (CRIC). The exclusion criteria were: the use of antipsychotics and neuroleptics and severe hypovisus. Patients were divided in 8 small groups matched for age, educational level and cognitive faculties. Each group underwent a rehabilitation cycle of visual arts mediated CAT.

Study participants underwent neuropsychological and functional assessments using standardized tests administered by expert neuropsychologists and speech therapists at baseline and at the end of treatment. In addition, participants and their partners completed an *ad hoc* structured questionnaire.

Fasting blood samples were also collected in vacutainer K3EDTA tubes and delivered to our Genomics and Environmental Mutagenesis Laboratory (Department of Cardiac, Thoracic, and Vascular Sciences and Public Health, University-Hospital of Padua) for biological aging assessment. During the enrolment period, each patient was carefully characterized by collecting data on demographics, education, smoking, Apolipoprotein E (APOE) genotyping, and basic biochemistry. The local Ethics Committee for Clinical Trials of the Province of Padova approved the study protocol (code number 3843/AO/16 and 3054/AO/14) in accordance with principles of the Helsinki Declaration. All subjects gave written informed consent and the study was conducted in accordance with the Declaration of Helsinki.

### 2.2 Visual arts mediated CAT protocol

CAT protocol, a non-pharmacological therapy for the improvement of functional status in patients with neurocognitive disorders, was developed by CRIC (Gollin et al., 2012). The treatment consisted in 17 sessions. 14 CAT sessions were performed at CRIC bi-weekly, including 1 h of physical activity led by the physiotherapist and 2 h of visual arts mediated CAT, in small groups, conducted by the speech therapists with the collaboration of students in training from the Department of Cultural Heritage of University of Padua as art experts. The other 3 CAT sessions, lasted 1 h and half, were carried out at the Eremitani Civic Museum in Padova.

### 2.3 Cognitive assessment

The Mini-Mental State Examination (MMSE) was used to obtain an index of global cognitive functioning (range 0–30) (Folstein et al., 1975). The Clock Drawing test was used to obtain a nonverbal index of global cognitive functioning (range 0–20) (Mendez et al., 1992).

The Digit Span (Wechsler, 1955) and the Corsi test was used to testing verbal and spatial short-term memory (Milner, 1971; Monaco et al., 2013). The Digit Span Backward and the Corsi Backward was used to testing verbal and spatial working memory (Monaco et al., 2013).

The Attentional Matrices (AM) (Spinnler and Tognoni, 1987) test was used to evaluate selective attention during a visual search, investigating different aspects of visual attention: speed and accuracy; the Trail Making Test (part A and B) (Giovagnoli et al., 1996) measured selective, sustained and divided attention.

The Visual Object and Space Perception (VOSP) battery was performed to assess object and space perception (Giovagnoli et al., 1996). The Copy of complex figure test [Rey–Osterrieth (Bertolani et al., 1993) and Taylor Figure (Casarotti et al., 2014)] was administered to evaluate constructive apraxia.

Since age and education have an influence on the distribution of cognitive performance, and in order to compare subjects with different anthropometric characteristics, the raw scores (PG) of some tests (i.e., MMSE, Digit Span Forward, Forward Corsi, AM, Trial making test (A, B, B – A), Digit Span backward, Backward Corsi and Copy of complex geometric figure) were adjusted according to the relative influences of these variables. We applied the appropriate adjustment coefficient according to the correction tables for each test, as previously described (Spinnler and Tognoni, 1987; Bertolani et al., 1993; Giovagnoli et al., 1996; Magni et al., 1996; Monaco et al., 2013; Casarotti et al., 2014). We transformed the raw score (PG) into the correct score (PC) using the adjustment coefficients that were calculated on the basis of a non-linear multiple regression model by subtracting the mean score of the whole sample from the predicted values for each age and education class (Spinnler and Tognoni, 1987; Bertolani et al., 1993; Giovagnoli et al., 1996; Magni et al., 1996; Monaco et al., 2013; Casarotti et al., 2014).

### 2.4 Functional assessment

The Italian version of the Direct Functional Status (DAFS) was used to directly assess the ability to perform complex activities of daily living (Zanetti et al., 1998).

Functional communication skills were assessed by Communication Activities of Daily Living—Second edition (CADL-2) (Carlomagno et al., 2013).

### 2.5 Mood state evaluation

Depression was assessed with the Geriatric Depression Scale (GDS 15 items version) (Smarr and Keefer, 2011), which is a self-rating screening tool to measure depressive symptoms in older adults.

The Quality of life – AD questionnaire was used to estimate different aspects of quality life of patients (Logsdon et al., 2002).

### 2.6 DNA extraction from blood samples

The QIAamp DNA Mini Kit (Qiagen, Milan, Italy) was used to extract DNA from whole blood on a QIAcube System (Qiagen, Milan, Italy) for automated high-throughput DNA purification, according to a customized protocol (Campisi et al., 2021). In particular, 400 μL of whole blood from each sample was specifically processed for DNA extraction. The QIAxpert Quantification System (Qiagen, Milan, Italy) was used to measure DNA and evaluate its quality.

### 2.7 LTL analysis

LTL was assessed on genomic DNA using quantitative real-time PCR (qPCR) as previously described (Pavanello et al., 2017, 2020). By comparing the ratio of the number of telomere repeat copies (T) to single nuclear copies (S) in experimental samples to the T/S ratio of a pooled reference sample, this assay measures relative TL in genomic DNA. Human (beta) globin (hbg) was used as the single-copy gene. A “six-point” standard curve was generated from a serially diluted pool of DNA (obtained from DNA samples randomly selected in this study) ranging from 40 to 1.25 ng/μl in each plate to determine the relative amounts of T and S (in nanograms). PCR runs were conducted in triplicate on a StepOnePlus real-time PCR system (Applied Biosystems, Milan, Italy) and statistical analyses were performed using the average of the three T/S ratio measurements. A measurement was considered acceptable if the standard deviation (SD) between triplicate measurements was <25%. To test the reproducibility of the LTL measurements, we repeated the analysis on different days on the 58% of the total; the resulting interclass correlation coefficient (ICC), an indicator of measurement reliability, was 0.91, indicating excellent reliability.

### 2.8 DNAmAge analysis

DNAmAge was determined by analyzing the methylation levels of five selected markers (ELOVL2 C1orf132, KLF14, TRIM59 and FHL2) in genomic DNA using bisulfite conversion and Pyrosequencing methodology as previously described (Pavanello et al., 2022; Campisi et al., 2023), with some modifications to allow us to set up an almost entirely automated method by using the PyroMark Q48 Autoprep Autoprep (Qiagen, Milano, Italy). The resulting pyrograms generated by the instrument were automatically analyzed using the Pyromark Q48 Autoprep software (Qiagen, Milan, Italy). The methylation percentages of the methylated cytosines at the 5 CpG sites were entered into an online calculator system accessible at www.agecalculator.ies.krakow.pl, to estimate of biological age from DNA methylation analysis. Twenty percent of the samples were analyzed in two different days to verify the reproducibility of our results and the coefficient of variation (CV) in replicate pyrosequencing runs was 1.7%.

### 2.9 Sample size estimation

The sample size for a before-and-after study (Paired T-test), was calculated on the base of data obtained from our previous study (Pavanello et al., 2019) on patients after myocardial infarction, by using the StastDirects software. In particular, since the telomere analysis represents the limiting analysis, considering a difference in LTL of −0.24 (SD 0.14), 9 subjects are required to achieve 90% power of detecting the association with an alfa error ≤ 1%.

### 2.10 Statistical analysis

All statistical analyses were performed using StatsDirect 3 software, STATA 17.0, and Prism Software (version 9.0). Data are expressed as interval variables (mean ± SD) and categorical variables (number, %), at both the baseline (T0) and the follow-up (T1).

For paired samples, the chi-square test and the paired *t*-test were used to compare the two groups. Correlation was evaluated by simple linear regression models in order to provide a measure of the strength of dependence between two variables. Multiple Linear Regression was used to evaluate the influence of independent variables such as age, gender, education and days of treatment on both LTL and DNAmAge (dependent variables) of all study subjects. Results were considered significant when a value of *p* of ≤ 0.05 was obtained.

## 3 Results

### 3.1 Study population

Table 1 summarizes characteristics of the study population, including demographics, blood parameters at baseline, and treatments, which consists of 20 patients with mild to moderate neurocognitive disorders.

**Table 1:** Main characteristics of *n* = 20 patients at the baseline (T0), including interval variables (mean ± SD), categorical variables (number, %) and cut-off for blood parameters values.

Variables	T0	Cut-off
*Demographic data*		
Age (years)	77.8 (±6.2)	
Sex		
Male (n, %)	11 (55%)	
Female (n, %)	9 (45%)	
Education (years)	10.3 (±4.67)	
Physical activity (n, %)	19 (96.8%)	
Diagnosis		
MCI (n, %)	5 (25%)	
AD (n, %)	7 (35%)	
LBD (n, %)	1 (5%)	
MD (n, %)	3 (15%)	
FTD (n, %)	1 (5%)	
PPA (n, %)	1 (5%)	
NPH (n, %)	1 (5%)	
TBI (n, %)	1 (5%)	
*Blood parameters*		
Leukocytes (10^3^/ml)	6.26 (±2.44)	4.40–11.00
Blood red cells (10^3^/ml)	4.36 (±0.61)	4.31–5.10
Hemoglobin (g/dl)	128.13 (±25.23)	123–153
Platelet count (10^3^/ml)	235.12 (±67.61)	150–450
Neutrophils (10^3^/ml)	3.68 (±1.78)	1.80–7.80
Lymphocytes (10^3^/ml)	1.79 (±0.53)	1.10–4.80
Monocytes (10^3^/ml)	0.58 (±0.29)	0.20–0.96
Eosinophils (10^3^/ml)	0.20 (±0.14)	0.00–0.50
Basophils (10^3^/ml)	0.04 (±0.07)	0.00–0.20
Cholesterol (mg/dl)	166.65 (±46.27)	
Triglycerides (mg/dl)	94.82 (±32.01)	
LDL (mg/dl)	91.69 (±44.32)	
HDL (mg/dl)	60.06 (±18.26)	
Glycated hemoglobin (mmol/mol)	42.27 (±7.58)	
Creatinine (mg/dl)	0.87 (±0.22)	
Urea (mmol/L)	6.13 (±2.73)	2.50–7.50
Bilirubin (mg/dl)	0.04 (±0.18)	/
Total protein	71.14 (±13.03)	64–83
Aspartate aminotransferase (U/L)	24.12 (±4.96)	10–35
Homocisteine (u-mol/L)	12.73 (±2.67)	0.00–15.00
*APOE genotyping (SNPs)*		
E3/E3 (n, %) (1)	10 (62.5%)	
E3/E4 (n, %) (2)	3 (18.75%)	
E2/E3 (n, %) (3)	0 (0%)	
E2/E4 (n, %) (4)	1 (6.25%)	
E4/E4 (n, %) (5)	2 (12.5%)	
*Treatments*		
*Cognitive activation therapy (CAT)*		
Days of treatment	43.45 (±12.1)	
Hours of treatment	30.03 (±3.49)	
*Physical activity*		
Days of treatment	8.4 (±3.28)	
Hours of treatment	8.4 (±3.28)	

AD, Alzheimer’s disease; MCI, Mild Cognitive Impairment; LBD, Lewy body dementia; MD, Mixed dementia; FTD, Frontotemporal degeneration; PPA, Primary progressive aphasia; NPH, Normal Pressure hydrocephalus; TBI, Traumatic brain injury; HDL, High density lipoprotein; LDL, Low density lipoprotein.

### 3.2 Cognitive and functional assessment, and mood state evaluation

Table 2 shows the results of the cognitive and functional assessments, as well as mood state evaluations conducted at baseline and post-treatment. Among the cognitive tests, significant score improvements were observed for “Copy of complex geometric figure” (PG and PC, *p* < 0.0001), some subtests of the VOSP including “Progressive Silhouettes” (p < 0.0001), “Position Discrimination” (*p* = 0.0349), and “Object decision” (*p* = 0.0594). Functional tools, including CADL-2 (*p* < 0.0001) and DAFS (*p* < 0.0001), also exhibited significant score increase.

**Table 2:** Scores and results of all cognitive and functional tests and mood state tools performed by *n* = 20 patients at baseline (T0) and after treatment at the follow up (T1), including interval variables (mean ± SD), categorical variables (number, %), cut-off, and *p*-values.

Variables	T0	T1	Cut-off PC	*P*
*Cognitive test/tools*				
*Screening*				
Mini mental state examination (MMSE)				
PG	23.4 (±2.74)	24.63 (±2.81)		0.1031
PC	22.66 (±2.56)	23.86 (±2.35)	≤24	0.1666
Clock drawing test	16.4 (±2.21)	15.74 (±4.73)	≤18	0.421
*Short-term memory*				
Digit span forward				
PG	4.7 (±0.80)	4.97 (±0.81)		0.0551
PC	5.16 (±0.79)	5.33 (±0.85)	<4.26	0.0551
Pathologic (n, %)	2 (10%)	0 (0%)		
Borderline (n, %)	2 (10%)	2 (10.53%)		
Non pathologic (n, %)	16 (80%)	17 (89.47%)		
Forward corsi				
PG	4.47 (±1.12)	4.71 (±1.16)		0.5292
PC	4.95 (±1.17)	5.20 (±1.19)	<3.46	0.5292
Pathologic (n, %)	1 (5%)	0 (0%)		
Borderline (n, %)	5 (25%)	1 (5.88%)		
Non pathologic (n, %)	14 (70%)	16 (84.21%)		
*Attentional skills*				
Attentional matrices				
PG	43.75 (±9.65)	45.95 (±9.54)		0.3418
PC	45.15 (±9.99)	47.46 (±10.73)	≤30	0.3418
Pathologic (n, %)	1 (5%)	2 (10.52%)		
Borderline (n, %)	1 (5%)	0 (0%)		
Non pathologic (n, %)	18 (90%)	17 (89.47%)		
Trial making test A				
PG	74.5 (±32.52)	68.05 (±25.60)		0.2746
PC	43.2 (±38.02)	36 (±34.78)	≥94	0.2464
Pathologic (n, %)	1 (5%)	0 (0%)		
Borderline (n, %)	2 (10%)	3 (15.79%)		
Non pathologic (n, %)	17 (85%)	16 (84.21%)		
Trial making test B				
PG	278.29 (±98.36)	289.06 (±80.36)		0.5577
PC	199.82 (±103.97)	207.5 (±87.29)	≥283	0.5888
Pathologic (n, %)	3 (17.65%)	3 (16.67%)		
Borderline (n, %)	7 (41.18%)	7 (38.89%)		
Non pathologic (n, %)	7 (41.18%)	8 (44.44%)		
Trial making test B – A				
PG	206.59 (±84.43)	220.28 (±80.56)		0.406
PC	157.35 (±79.73)	169.61 (±77.23)	≥187	0.406
Pathologic (n, %)	8 (47.06%)	8 (44.44%)		
Borderline (n, %)	2 (11.76%)	3 (16.67%)		
Non pathologic (n, %)	7 (41.18%)	7 (38.89%)		
*Working memory*				
Digit Span backward				
PG	3.48 (±0.63)	3.41 (±0.95)		0.6499
PC	3.86 (±0.75)	3.81 (±1.11)	<2.65	0.6499
Pathologic (n, %)	1 (5%)	1 (5.26%)		
Borderline (n, %)	3 (15%)	4 (21.05%)		
Non pathologic (n, %)	16 (80%)	15 (78.95%)		
Backward corsi				
PG	4 (±1.11)	4 (±0.89)		0.5445
PC	4.55 (±1.18)	4.57 (±0.98)	<3.17	0.6065
Pathologic (n, %)	3 (15%)	2 (12.50%)		
Borderline (n, %)	1 (5%)	0 (0%)		
Non pathologic (n, %)	16 (80%)	14 (87.50%)		
*Constructive skills*				
Copy of complex geometric figure				
PG	28.26 (±4.41)	31.68 (±4.19)		**<0.0001**
PC	28.50 (±4.09)	33.48 (±4.05)	pre < 28; post <27.66	**<0.0001**
Pathologic (n, %)	8 (42.11%)	1 (5.26%)		
Borderline (n, %)	7 (36.84%)	2 (10.53%)		
Non pathologic (n, %)	4 (21.05%)	16 (84.21%)		
*Visual-perceptual/visual–spatial skills*				
Visual object and space perception battery (VOSP)				
Screening	19.25 (±0.72)	19.53 (±0.70)	≤15	0.2623
Pathologic (n, %)	0 (0%)	0 (0%)		
Borderline (n, %)	0 (0%)	0 (0%)		
Non pathologic (n, %)	20 (100%)	19 (100%)		
Incomplete letters	18.1 (±1.29)	18.78 (±1.39)	≤17	0.0723
Pathologic (n, %)	2 (10%)	1 (5.26%)		
Borderline (n, %)	0 (0%)	0 (0%)		
Non pathologic (n, %)	18 (90%)	18 (94.74%)		
Silhouettes	14.9 (±4.63)	15.47 (±4.45)	≤16	0.6416
Pathologic (n, %)	9 (45%)	8 (42.11%)		
Borderline (n, %)	2 (10%)	1 (5.26%)		
Non pathologic (n, %)	9 (45%)	10 (52.63%)		
Object decision	14.4 (±3.70)	16.05 (±2.07)	≤15	**0.0594**
Pathologic (n, %)	6 (30%)	2 (10.53%)		
Borderline (n, %)	3 (15%)	2 (10.53%)		
Non pathologic (n, %)	11 (55%)	15 (78.95%)		
Progressive silhouettes	12.25 (±2.99)	11.05 (±2.53)	≥14	**<0.0001**
Pathologic (n, %)	2 (10%)	0 (0%)		
Borderline (n, %)	1 (5%)	2 (10.53%)		
Non pathologic (n, %)	17 (85%)	17 (89.47%)		
Dot	9.9 (±0.31)	9.63 (±0.76)	≤ 8	0.1105
Pathologic (n, %)	0 (0%)	1 (5.26%)		
Borderline (n, %)	0 (0%)	0 (0%)		
Non pathologic (n, %)	20 (100%)	18 (94.74%)		
Position discrimination	18.95 (±1.39)	19.53 (±0.77)	≤18	**0.0349**
Pathologic (n, %)	4 (20%)	1 (5.26%)		
Borderline (n, %)	2 (10%)	0 (0%)		
Non pathologic (n, %)	14 (70%)	17 (89.47%)		
Number Location	8.47 (±2.03)	8.53 (±2.01)	≤7	0.848
Pathologic (n, %)	4 (21.05%)	3 (15.79%)		
Borderline (n, %)	1 (5.26%)	1 (5.26%)		
Non pathologic (n, %)	14 (73.68%)	15 (78.95%)		
Cube analysis	8.5 (±2.06)	8.84 (±1.26)	≤6	0.461
Pathologic (n, %)	3 (15%)	3 (15.79%)		
Borderline (n, %)	0 (0%)	1 (5.26%)		
Non pathologic (n, %)	17 (85%)	15 (78.95%)		
*Mood state*				
Geriatric depression scale (GDS)	3.4 (±3.10)	3.26 (±2.28)	≥6	0.6875
Quality of life (QoL)				
Patients	34.75 (±6.16)	35.26 (±6.30)		0.2791
Caregivers	39.0 (±5.24)	39.06 (±4.11)		0.6215
*Functional test/tools*				
*Functional communication*				
Communication activities of the daily living (CADL 2)	88.71 (±5.17)	95.68 (±4.03)		**<0.0001**
*Direct assessment of functional status*				
Direct assessment of functional status (DAFS)	67.25 (±6.77)	75.79 (±6.07)	≤68	**<0.0001**

PC, correct score; PG, raw score.

### 3.3 LTL

**Figure 2:**
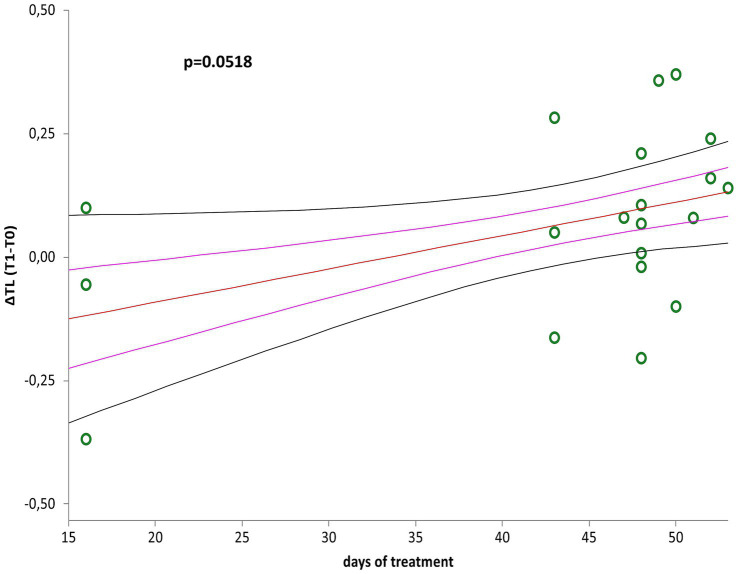
Correlation curve between ∆LTL (∆LTL = LTL T1–LTL T0) and days of treatment of patients with mild to moderate neurocognitive disorders (*n* = 20). A simple linear regression plot shows the correlation between ∆LTL and days of treatment [correlation coefficient (r) = 0.440705; two-sided *p* = 0.0518]. Mean, standard error (SE), and 95% coefficient intervals (CI) are represented as orange, pink, and black lines, respectively.

**Figure 3:**
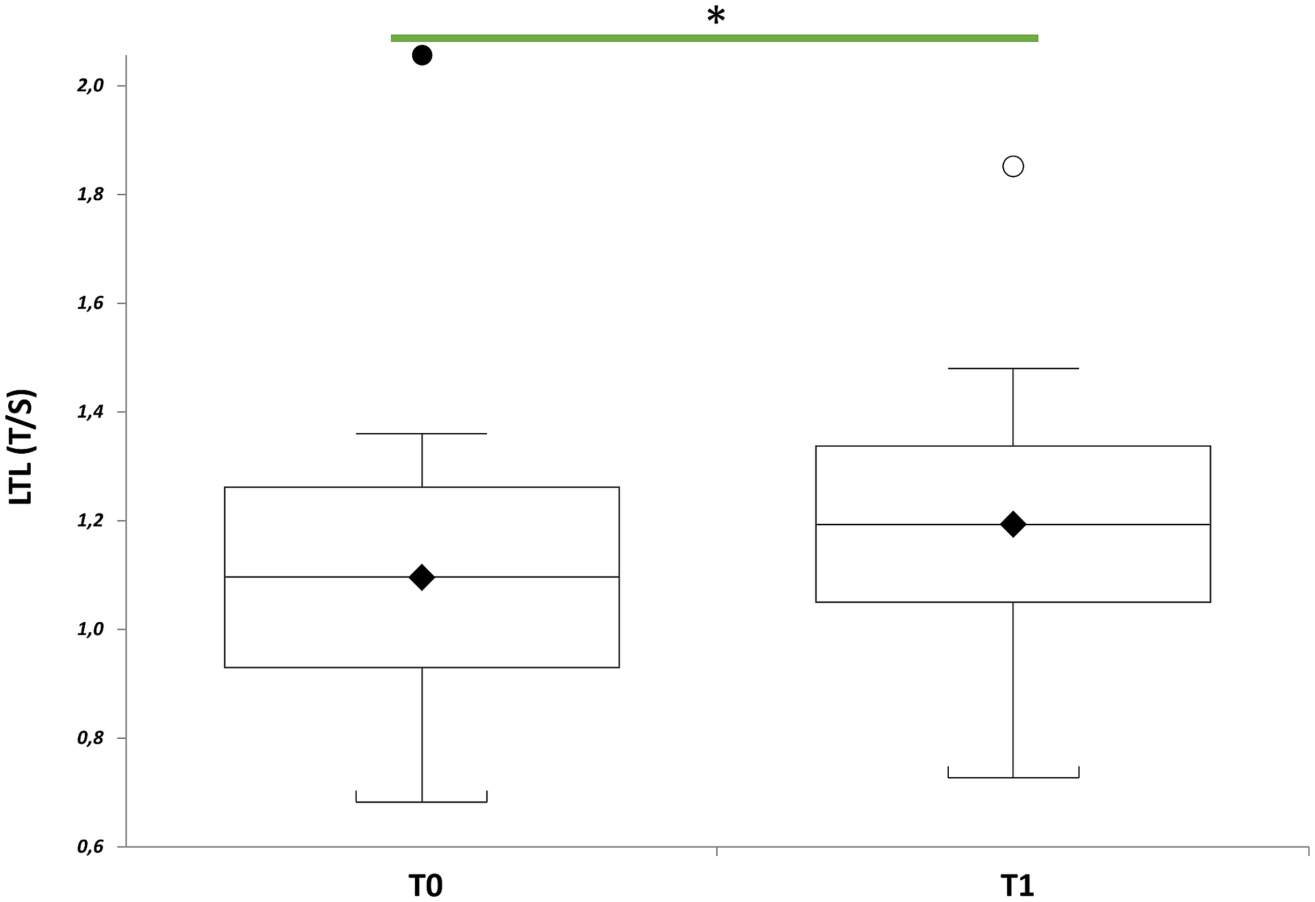
LTL of patients with mild to moderate neurocognitive impairments receiving visual arts-mediated Cognitive Activation Therapy (CAT). Box plots show levels of LTL in patients with mild to moderate neurocognitive impairments before (T0) and after CAT (T1) (*n* = 20). In box plots, the boundary of the box closest to the *x*-axis indicates the 25th percentile, the line within the box marks the mean, and the boundary of the box farthest from the *x*-axis indicates the 75th percentile. Whiskers (error bars) above and below the box indicate the 95 and 5th percentiles. The horizontal bar with one asterisk indicates the significant comparison between T0 and T1 LTL of the same patient (*n* = 20) (T1 vs. T0, paired *t*-test: mean 1.19 ± 0.28 T/S vs. 1.09 ± 0.32 T/S, *p* = 0.0269).

∆LTL (∆LTL = LTL T1- LTL T0) positively correlated with days of treatment (Figure 2, *r* = 0.44; *p* = 0.0518) when considered the whole group (*n* = 20), although LTL remained relatively stable after treatment (*p* = 0.1248). However, LTL increased significantly considering those (*n* = 17) participating the entire period of therapy (mean of 43 days), and excluding the three participants who underwent only 16 days of treatment (Figure 3, *p* = 0.0269).

Stratifying the study population by sex (Table 3), revealed that men had significantly shorter LTL than women at baseline (*p* = 0.0012), but not at post-treatment (*p* = 0.1688). LTL, in fact, increased significantly in men (*p* = 0.0142), but not in women (*p* = 0.578), after therapy. Multiple linear regression analysis within the group of seventeen patients (Table 4) revealed correlations between ∆LTL and age (*p* = 0.0357), male gender (*p* = 0.025) and education level (*p* = 0.0008), and not with days of treatment (*p* = 0.4812).

**Table 3:** LTL (mean ± SD) of *n* = 20 patients, divided into two groups by sex, at baseline (T0) and after treatment (T1), and the p-values comparing the measurements in both groups.

	T0	T1	p*
*Men*	0.95 ± 0.18	1.10 ± 0.24	**0.0142**
*Women*	1.32 ± 0.30	1.28 ± 0.27	0.578
*P^$^*	**0.0012**	0.1688	

*Paired *t* tests two sided. ^$^Mann–Whitney U test two sided.

**Table 4:** Multiple regression analysis of the influence of age, gender, education (years) and days of treatment on ∆LTL.

	*b*	*r*	*t*	*p*
*Age*	b1 = −0.011079	*r* = −0.563855	*t* = −2.365074	***p* = 0.0357**
*Sex (M = 1; F = 2)*	b2 = −0.133792	*r* = −0.594421	*t* = −2.560623	***p* = 0.025**
*Education*	b3 = 0.026492	*r* = 0.787385	*t* = 4.424574	***p* = 0.0008**
*Days of treatment*	b4 = 0.006236	*r* = 0.2054	*t* = 0.72703	*p* = 0.4812

Simple linear regression analyses indicated no correlation between LTL at baseline (T0) or post-treatment (T1) and chronological age (Supplementary Figure 1A, *p* = 0.7552; Supplementary Figure 1B, *p* = 0.9908). Moreover, multiple linear regression analysis showed that shorter LTL at baseline in men (Supplementary Table 1) correlated with increased monocyte counts (*p* = 0.0409), but not with the other variables considered, including total leukocyte, neutrophil and lymphocyte counts (*p* > 0.05). In contrast, no association was found in women (Supplementary Table 2, *p* > 0.05).

### 3.4 DNAmAge

**Figure 4:**
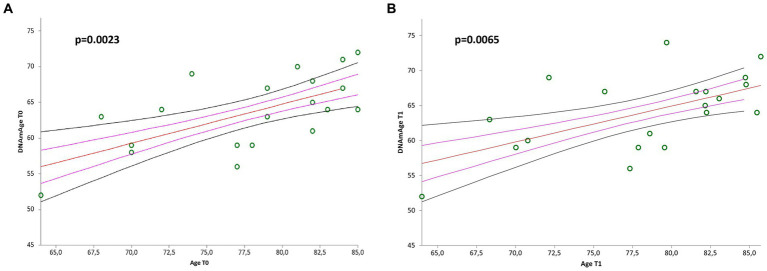
Correlation curves between DNAmAge and chronological age of *n* = 20 patients with mild to moderate neurological disorders before **(A)** and after **(B)** visual arts-mediated CAT. In **(A)**, a simple linear regression plot shows the correlation between DNAmAge and chronological age before treatment [correlation coefficient (r) = 0.641485; two-sided *p* = 0.0023]; while in **(B)**, simple linear regression plot showing the correlation between DNAmAge and chronological age after treatment [correlation coefficient (r) = 0.587001; two-sided *p* = 0.0065]. Mean, standard error (SE), and 95% coefficient intervals (CI) are represented as orange, pink, and black lines, respectively.

Simple linear regression analyses showed positive correlation between DNAmAge, at both baseline (T0) and at end of treatment (T1), and chronological age (Figure 4A, *r* = 0.641485, *p* = 0.0023; Figure 4B, *r* = 0.587001, *p* = 0.0065).

In Table 5, DNAmAge did not change significantly after treatment (*p* = 0.3622), even though we excluded three participants who underwent 16 days of treatment (*p* = 0.4624). Moreover, ∆DNAmAge (∆DNAmAge = DNAmAge T1- DNAmAge T0) showed no correlation with days of treatment (Supplementary Figure 2, *p* = 0.7133). Furthermore, multiple linear regression analysis for ∆DNAmAge (Supplementary Table 3) found no significant correlations with age, sex, education level, or days of treatment (*p* > 0.05). No correlation was found between differences in DNAmAge and in LTL (Supplementary Figure 3, *p* = 0.5599).

**Table 5:** DNAmAge (mean ± SD) of *n* = 20 and *n* = 17 patients (excluding three patients who underwent CAT for 16 days) at baseline (T0) and after treatment (T1), and the p-values comparing the measurements in both groups.

N	T0	T1	p*
*20*	63.55 ± 5.31	64.5 ± 5.48	0.3622
*17*	64.12 ± 5.46	64.59 ± 5.72	0.4624

*Paired *t* tests two sided.

### 3.5 Association between LTL measures of biological aging and cognitive and functional status

No associations were observed at baseline (T0) and follow-up (T1) (*p* > 0.05) between LTL measures and cognitive and functional screening test scores which resulted significantly changed after treatment (Supplementary Table 4). Supplementary Figure 4 shows no correlation between ∆LTL and these cognitive performance test measures (*p* > 0.05).

## 4 Discussion

This study represents the first attempt to integrate visual arts-mediated CAT with biomarkers of biological aging in patients experiencing neurocognitive disorders in the mild to moderate phase.

Our study reveals that:

1. LTL elongates significantly after the entire visual arts-mediated CAT (mean of 43 days), specifically in men who seem to greater benefit from therapy than women. Furthermore, LTL elongation in all subjects correlated to days of treatment. Determinants of LTL elongation were age (being younger), male sex, as well as education level. No influence of treatment on the DNAmAge was observed.
2. Several cognitive aspects related to visuo-constructional ability, short memory, object and space perception, functional communication skills and functional capabilities improved after visual arts-mediated CAT. LTL elongation did not correlate with improvements in cognitive and functional tests.

Our finding in LTL elongation after the entire visual arts-mediated CAT treatment is in line with a previous study by Mahendran et al. (2018), who reported an increase in LTL in subjects with mild cognitive impairment after 9 months of art therapy involving the physical creation of themed artwork. Our result is also coherent with a previous study that explored the beneficial effect of meditation and music listening on LTL of adults with subjective cognitive decline (Innes et al., 2018). As suggested in our previous work (Pavanello et al., 2019), the mechanism hypothesis is that relaxing practices, by inducing the so-called relaxation response (RR), counteract psychological stress, causing favorable changes in some inflammatory genes and stress hormones, via a cascade of neuroendocrine-immune messengers, and improvements in endothelial function hormones (Dal Lin et al., 2018). This reduces not only oxidative stress and inflammation, but also glucocorticoids, the major outcome of the hypothalamic–pituitary–adrenal axis by stress (Munhoz et al., 2006, 2010). Influential stress mediators including oxidative stress, inflammation, and glucocorticoids mediate telomere shortening, likely through inhibiting telomerase activity, the enzyme responsible for telomere length maintenance (de Punder et al., 2019; Lin and Epel, 2022). This hypothesis aligns with a previous work suggesting that reducing oxidative stress and inflammation promotes telomere lengthening (Kiecolt-Glaser et al., 2013). Conklin et al. (2018), who carried out an intensive meditation training in healthy subjects, reported that LTL elongated from both telomerase-independent and telomerase-mediated pathways. Innes et al. (2018) found that meditation increases LTL by activating telomerase (Zvereva et al., 2010).

Furthermore, engagement in art activities has been reported to be associated with increased serotonin activity (Mercer et al., 2010). As proposed by Bersani et al. (2015), an increase in serotonin levels could secondarily increase Telomerase Reverse Transcriptase (TERT) expression and telomerase activity through PI3K/Akt signaling. In turn, higher telomerase activity could lead to telomere elongation, bolster mitochondrial health, and induce growth factors that promote neurogenesis, and, potentially improve mental well-being (Epel and Prather, 2018). This potential link between serotonin and TERT expression through activation of the PI3K/Akt signaling pathway may be the same as that for antidepressants, such as lithium and certain antipsychotics (Kitagishi et al., 2012), which have also been found to increase LTL (Martinsson et al., 2013; Squassina et al., 2016; Coutts et al., 2019).

Stratifying by sex, we found that men had shorter LTL than women at baseline and LTL elongated after therapy only in men. Men therefore seem to respond better to this non-pharmacological treatment. The hypothesis is that reduction in chronic stress and the consequent reduction in oxidative stress could induce an increase in testosterone levels (MacLean et al., 1997) that may have direct and indirect (via conversion to oestradiol) effects to promote the expression and activity of telomerase, as shown by *in vitro* studies (Kyo et al, 1999; Nanni et al., 2002; Calado et al., 2009; Nourbakhsh et al., 2010). Similarly, treatment with danazol, a non-aromatisable androgen, used as treatment resulted in LTL elongation of patients with telomeric diseases (Townsley et al., 2016). Furthermore, telomere length is sex-specific, with girls having longer telomeres than boys (Factor-Litvak et al., 2016; Jylhävä et al., 2017) which persists throughout life (Gardner et al., 2014). Men effectively live shorter than women (Zhao and Crimmins, 2022) and the cellular and molecular mechanisms of aging are better preserved in women (Hägg and Jylhävä, 2021). The biological reason is related to estrogens’ activity that induces the production of telomerase (Nawrot et al., 2004) protecting against damage caused by reactive oxygen species (Aviv, 2002).

Lastly, since the anti-aging gene Sirtuin 1 (Sirt 1) is a NAD(+)-dependent class III histone deacetylase that plays a fundamental role in regulating LTL (Amano and Sahin, 2019), and considering that we have observed that the Visual Arts-Mediated CAT effectively reduces cellular aging in men by elongating LTL, it is plausible that plasma levels of Sirtuin 1 may be increase with Visual Arts-Mediated CAT and its measurement could play an important role in validating CAT as an early biomarker of cellular aging. Furthermore, the dietary interventions with Sirtuin 1 activators (Martins, 2016) together with visual arts-mediated CAT may accelerate the reduction in cellular aging by greater elongation of LTL. This hypothesis should be tested in future study.

Furthermore, in a multivariate analysis that considered the effect of leukocyte, neutrophil, lymphocyte and monocyte counts at baseline on LTL in men, only the monocyte count was significant. This finding is in line with a previous study that found a significant reduction in LTL monocytes in patients with AD or mild cognitive impairment compared to healthy people (Hochstrasser et al., 2012).

Determinants of LTL elongation after treatment were not only to be male, but also being younger and with higher education level. Middle-aged individuals tend to experience a slower rate of aging compared to older individuals (Berglund et al., 2016), which may be attributed to a greater reserve of cells and more receptive messengers susceptible to the elongation effect. They had a more significant elongation effect with a similar number of treatment days. Additionally, increased exposure to chronic stress is associated with a higher rate of telomere attrition (Epel and Prather, 2018). This effect is likely more pronounced in older individuals who are more vulnerable to stress (Osmanovic-Thunström et al., 2015).

We also confirmed the positive association between LTL and years of education, as reported in our (Pavanello et al., 2021) and others’ previous studies (Steptoe et al., 2011; Surtees et al., 2012). Amin et al. (2021) estimated that an additional year of education is associated with 0.32 fewer years of telomere aging. Additionally, a higher level of education can play a significant role in helping people to appreciate visual art. A greater appreciation of the visual arts could lead to a greater participants engagement/emotional involvement of participants, greater awareness of the importance of treatment and greater adherence to the CAT protocol, resulting in significant benefits. de Medeiros et al. (2007), in an 8-week autobiographical writing workshop for a group of community-dwelling older adults, pointed out the importance of a high level of education to capture improvements.

Our study also showed that visual arts-mediated CAT had no effect on DNAmAge. This finding is in line with a systematic review that observed no association between DNAmAge and dementia or Alzheimer’s disease due to limitations of epigenetic aging clocks for dementia or cognitive aging biomarkers (Zhou et al., 2022).

Moreover, we did not find the significant inverse correlation between LTL and chronological age, that is well documented in the literature (Müezzinler et al., 2013). This could be due to the narrow age range. On the other hand, DNAmAge was highly correlated with chronological age, both before and after treatment, confirming the validation of the quality of our analysis and the predictive power of our model, with a mean deviation from chronological age comparable to the reference methods of Horvath (2013) and Hannum et al. (2013).

Visual arts-mediated CAT improved several cognitive aspects related to visuo-constructional ability, object and space perception, functional communication skills and functional capabilities. In particular, the “Copy of complex geometric figure” test showed a reduction in score after treatment in 7 patients, reducing the number of patients with a pathological score from 8 to 1. This revealed an improvement in visuo-constructional ability and visual memory after the visual arts-mediated CAT. Two subtests of the VOSP, i.e., progressive silhouettes and position, reported a decrease in the number of patients with a pathological score from 2 to 0 and from 4 to 1, respectively. These subtests, which assess object and spatial perception, suggested an improvement in the occipito-temporal and occipito-parietal neural circuits. We also found improvements in functional communication skills and general functional ability, as indicated by increases in the scores on the CADL-2 and DAFS tools. Improvements in cognitive functions is also found by Rawtaer et al. (2015) in a community-dwelling older people who participated in psychosocial interventions, including art therapy. Previous studies have reported a positive association between increased dopamine levels and artistic performance in patients with Parkinson disease (Walker et al., 2006; Kulisevsky et al., 2009; Schwingenschuh et al., 2010), suggesting that the opposite mechanism could also be plausible. Increased dopamine synthesis capacity in older adults has been found to reduce the impact of atrophy on cognitive decline, supporting a model by which upregulation of dopamine synthesis is a mechanism of cognitive resilience in aging (Ciampa et al., 2022). In summary our and the above studies suggest that the improvement in several cognitive aspects could be due to an increased dopamine synthesis capacity, confirming the importance in integrating non-pharmacological treatments in people with dementia to improve cognitive performance (Johnson et al., 2020). However, unlike to Mahendran et al. (2018), we did not find an improvement in Digit Span Forward or a decrease in GDS score, which are the common tests with our study. This could be due to the short duration of our study as well as the different intervention protocols, i.e., art therapy involving the physical creation of themed artwork versus visual arts-mediated CAT.

Improvements in cognitive and functional tests did not correlate with LTL elongation. This finding is in line with a previous randomized trial on art therapy and music reminiscence in older people with mild cognitive impairment (Mahendran et al., 2018). However, another randomized trial, conducted in subjects with cognitive decline underwent meditation and music listening, reported an association between increased LTL and improvements in cognitive function, sleep, mood, and quality of life, suggesting a potential functional relationship (Innes et al., 2018). The differences in results between our study and that of Innes et al. (2018) could be explained by heterogeneity in terms of variations in participants’ characteristics, differences in the interventions, and study duration. While our study population consisted in patients with mild to moderate neurocognitive disorders who underwent an entire cycle of visual art-mediated CAT for approximately 43 days; the population of Innes’s study (Innes et al., 2018) comprised adults with subjective cognitive decline who underwent an intervention of Kirtan Kriya meditation and music listening for 84 days.

This study is marked by certain limitations that warrant acknowledgment. Given its nature as a pilot study exploring the impacts of a tailored CAT protocol, it is important to note the small sample size. However, the sample size estimation reveals that it is sufficient to obtain statistically significant results and draw meaningful conclusions from the data collected. Furthermore, the absence of an age-matched control group is another notable constraint, as is the lack of analysis of telomerase expression or activity. Consequently, future research is imperative, and our forthcoming endeavors will focus on expanding the participant pool, with the inclusion of a well-matched control group, and the analysis of telomerase and plasma levels of Sirtuin 1, which plays a critical role in the aging process (Wątroba et al., 2017) as well as synaptic plasticity and cognitive function (Michán et al., 2010; Fagerli et al., 2022).

Moreover, the positive effect of visual art-mediated CAT on LTL but not on DNAmAge could be due to the fact that these biological indicators of aging respond to molecular mechanisms in completely different ways. In fact, we did not find any correlation between differences in LTL and in DNAmAge, and this result aligns with previous studies (Belsky et al., 2018; Marioni et al., 2018; Pavanello et al., 2019; Vyas et al., 2019). Despite the short time frame of our study, this timing choice was supported by our previous experience (Pavanello et al., 2019) and a thorough review of the existing literature (Figure 1), which showed that 6 of the 8 studies on positive effects on TL had a timeframe comprised between 1 and 3 months (Thimmapuram et al., 2017; Conklin et al., 2018; Innes et al., 2018; Tolahunase et al., 2018; Le Nguyen et al., 2019; Pavanello et al., 2019), while 2 out of the 4 studies on DNAmAge had a timeframe of 2 months (Pavanello et al., 2019; Fitzgerald et al., 2021). We therefore believe that this rationale provides a robust basis for the selected study. Furthermore, short-term treatment is the best choice to minimize study withdrawal and further bias.

Despite these limitations, the longitudinal design employed in this research stands out as a notable strength. Each participant underwent analysis both before and after engaging in CAT, effectively making each subject their own control. This approach allowed us to compare each individual’s pre- and post-intervention outcomes, thus minimizing inter-subject variability and providing a robust internal control mechanism. Additional strengths of our study include a meticulously structured study design, based on a customized visual arts-mediated CAT protocol. Furthermore, we employed validated measures encompassing indicators of biological aging and standardized cognitive and functional tools, adding robustness to our findings.

## 5 Conclusion

In an aging world, with a consequent increase in chronic diseases, in particular dementia and neurological disorders, the anti-aging strategies are of growing interest. Our study shows that modern cognitive neuroscience can contribute to well-being and health throughout the esthetic experience and enjoyment of beauty. This pilot study yielded promising results, indicating the efficacy of the non-pharmacological visual arts-mediated CAT on biological aging of patients with neurocognitive disorders, in particular slowing down the mitotic cellular aging, i.e., LTL, in men. This result could be attributed to the reduction of chronic stress and consequent decline in oxidative stress and inflammation through the induction of the RR linked to neuroendocrine regulation.

Integrating non-pharmacological treatments into dementia care is crucial for enhancing overall well-being. This research contributes to the evolving field of dementia care and may provide valuable insights into novel treatment approaches, although future longitudinal studies with larger populations and longer duration are necessary to validate and further elucidate these findings.

## Data availability statement

The raw data supporting the conclusions of this article will be made available by the authors, without undue reservation.

## Ethics statement

The local Ethics Committee for Clinical Trials of the Province of Padova approved the study protocol (code number 3843/AO/16 and 3054/AO/14) in accordance with principles of the Helsinki Declaration. All subjects gave written informed consent.

## Author contributions

MC: Writing – original draft, Writing – review & editing, Data curation, Formal analysis, Investigation, Methodology. LC: Data curation, Formal analysis, Investigation, Methodology, Writing – original draft, Writing – review & editing. DC: Data curation, Writing – review & editing. CG: Writing – review & editing, Conceptualization, Resources, Supervision. DG: Methodology, Writing – review & editing, Investigation. MS: Methodology, Writing – review & editing, Data curation. CR: Data curation, Formal analysis, Writing – review & editing, Investigation. EF: Formal analysis, Investigation, Writing – review & editing. SP: Resources, Writing – review & editing, Conceptualization, Funding acquisition, Project administration, Supervision, Visualization, Writing – original draft.
