# Supplementary material for: Mitigating cellular aging and enhancing cognitive functionality: visual arts-mediated Cognitive Activation Therapy in neurocognitive disorders
Source: Front Aging Neurosci. 2024 Mar 8;16:1354025. doi: 10.3389/fnagi.2024.1354025 (PMC10957554; doi:10.3389/fnagi.2024.1354025)
Supplement: Supplementary file 1 [file Data_Sheet_1.docx]

Supplementary Material

# Supplementary Figures and Tables

## Supplementary Figures

**
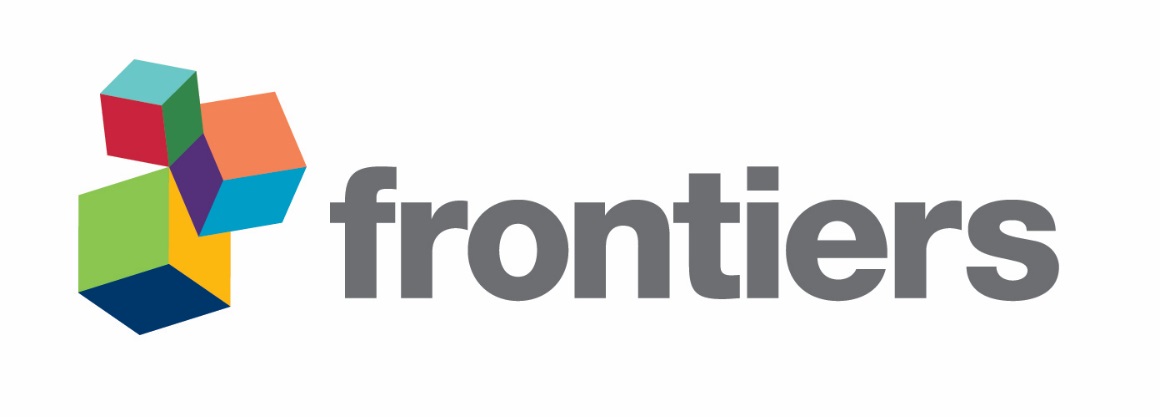
**

**Supplementary Figure 1.** **Correlation curves between LTL and chronological age of n =20 patients with mild to moderate neurological disorders before (A) and after (B) visual arts-mediated CAT.** In (A), a simple linear regression plot shows the correlation between LTL and chronological age before treatment [correlation coefficient (r)=0.07441; two-sided p=0.7552]; while in (B), simple linear regression linear regression plot showing the correlation between LTL and chronological age after treatment [correlation coefficient (r)=0.002759; two-sided p=0.9908]. Mean, standard error (SE), and 95% coefficient intervals (CI) are represented as orange, pink, and black lines, respectively.

**
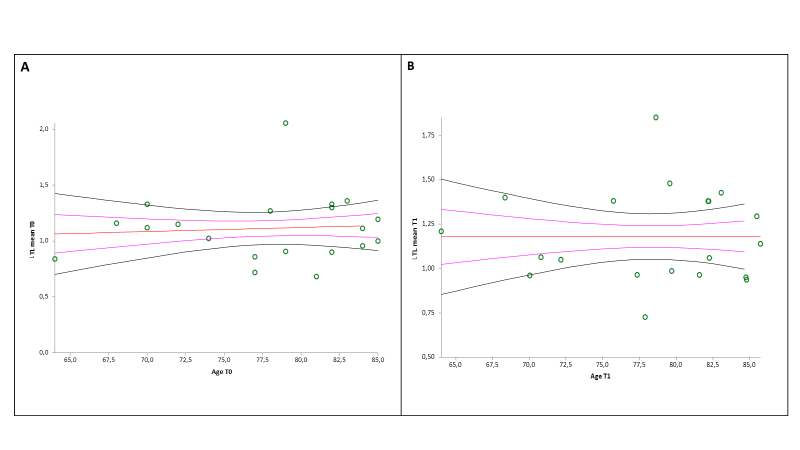
**

**Supplementary Figure 2.** **Correlation curve between** **∆DNAmAge (∆DNAmAge = DNAmAge T1- DNAmAge T0) and days of treatment of patients with mild to moderate neurocognitive disorders (n = 20).** A simple linear regression plot shows the correlation between ∆DNAmAge and days of treatment [correlation coefficient (r) =0.373308; two-sided p= 0.7133]. Mean, standard error (SE), and 95% coefficient intervals (CI) are represented as orange, pink, and black lines, respectively.

**
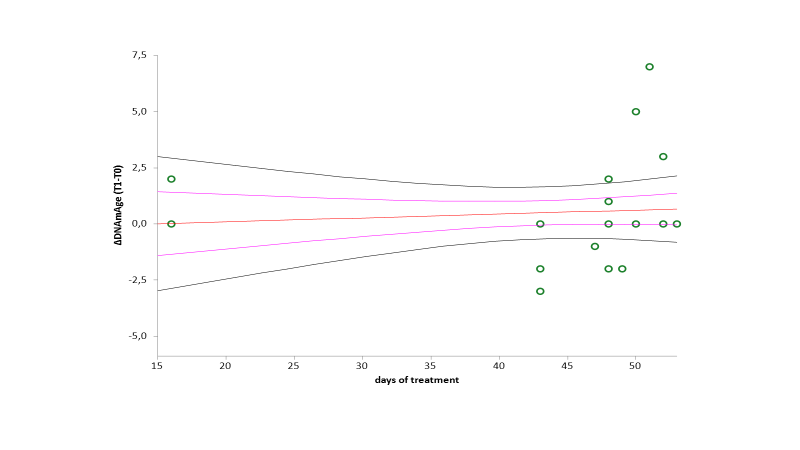
**

**Supplementary Figure 3. Correlation curve between** **∆DNAmAge and ∆LTL of patients with mild to moderate neurocognitive disorders (n = 20).** A simple linear regression plot shows the correlation between ∆DNAmAge and ∆LTL [correlation coefficient (r)=0.596247; two-sided p= 0.5599]. Mean, standard error (SE), and 95% coefficient intervals (CI) are represented as orange, pink, and black lines, respectively.

**
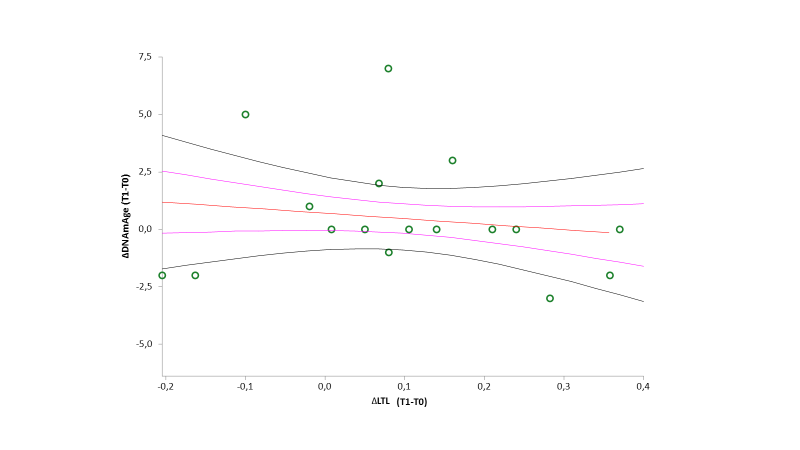
**

**Supplementary Figure 4. Associations between ∆LTL and seven cognitive performance test measures: Communication Activities of the Daily Living (CADL 2); Direct Assessment of Functional Status (DAFS); Visual Object and Space Perception Battery (VOSP): Obj Decision, Progressive Silhouettes, Position; Copy of complex geometric figure (Figure PG & PC).** Graphs indicate the associations between ∆LTL and seven cognitive performance test measures: Communication Activities of the Daily Living (CADL 2); Direct Assessment of Functional Status (DAFS); Visual Object and Space Perception Battery (VOSP): Obj Decision, Progressive Silhouettes, Position; Copy of complex geometric figure (Figure PG & PC). The cognitive function scores (x-axis) are divided into four quintiles; the black dots show the mean TL value, and the whiskers represent the 95% confidence interval. ∆LTL (TL T1-TL T0) can be seen on the y-axis. The associations between ∆LTL and differences in cognitive test scores were not statistically significant (p>0.05).


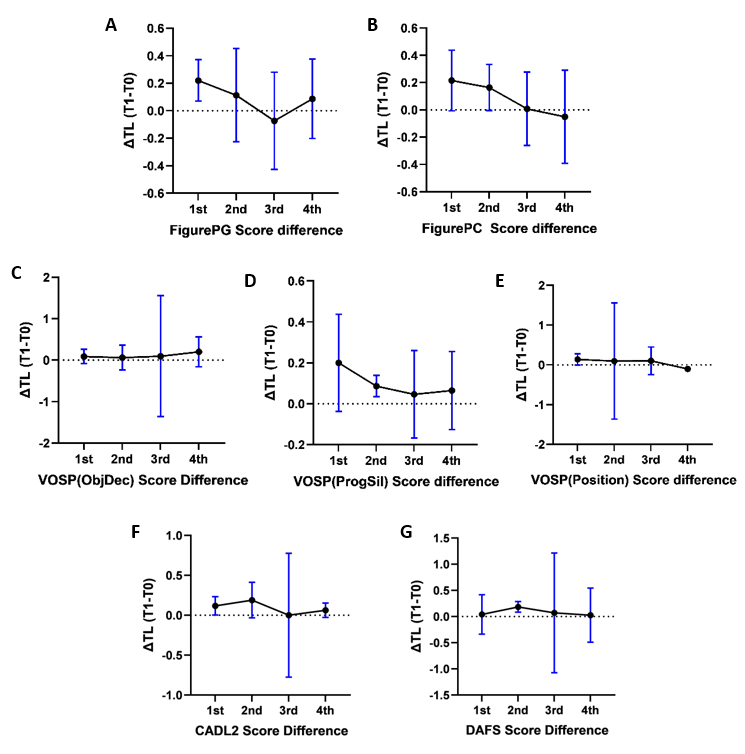


## Supplementary Tables

**Supplementary Table 1**. Multiple regression analysis of the influence of leukocytes count (10^3^/ml), neutrophils (10^3^/ml), lymphocytes (10^3^/ml) and monocytes (10^3^/ml) on TL at baseline (T0) in men.

|  | b | | r | | t | | p | |  |
| --- | --- | --- | --- | --- | --- | --- | --- | --- | --- |
| Leukocytes (10^3^/ml) | | b1 = 0.544888 | | r = 0.627391 | | t = 1.39549 | | p = 0.2572 | |
| Neutrophils (10^3^/ml) | | b2 = -0.487581 | | r = -0.548027 | | t = -1.134793 | | p = 0.3389 | |
| Lymphocytes (10^3^/ml) | | b3 = -0.222692 | | r = -0.280435 | | t = -0.506033 | | p = 0.6477 | |
| Monocytes (10^3^/ml) | | b4 = -1.195586 | | r = -0.893844 | | t = -3.452857 | | **p = 0.0409** | |

**Supplementary Table 2**. Multiple regression analysis of the influence of leukocytes count (10^3^/ml), neutrophils (10^3^/ml), lymphocytes (10^3^/ml) and monocytes (10^3^/ml) on TL at baseline (T0) in women.

|  | b | | r | | t | | p | |  |
| --- | --- | --- | --- | --- | --- | --- | --- | --- | --- |
| Leukocytes (10^3^/ml) | | b1 = 0.030017 | | r = 0.013139 | | t = 0.02628 | | p= 0.9803 | |
| Neutrophils (10^3^/ml) | | b2 = -0.051621 | | r = -0.021484 | | t = -0.042977 | | p = 0.9678 | |
| Lymphocytes (10^3^/ml) | | b3 = 0.054882 | | r = 0.023374 | | t = 0.046761 | | p = 0.9649 | |
| Monocytes (10^3^/ml) | | b4 = 0.357314 | | r = 0.096102 | | t = 0.193097 | | p = 0.8563 | |

**Supplementary Table 3**. Multiple regression analysis of the influence of age, gender, education (years) and days of treatment on ∆DNAmAge.

|  | b | r | t | p |
| --- | --- | --- | --- | --- |
| *Age* | b1 = -0.029586 | r = -0.079734 | t = -0.309797 | p = 0.761 |
| *Sex (M=1; F=2)* | b2 = 0.928394 | r = 0.184114 | t = 0.725473 | p = 0.4793 |
| *Education (years)* | b3 = -0.190832 | r = -0.35653 | t = -1.477962 | p = 0.1601 |
| *days of treatment* | b4 = 0.016637 | r = 0.077873 | t = 0.30252 | p = 0.7664 |

Supplementary Table 4. Associations between LTL measures and cognitive and functional screening tests that showed significant improvements, at baseline (T0) and after treatment (T1).

| **T0** | **LTL** | **T1** | **LTL** |
| --- | --- | --- | --- |
|  | **r (p-value)** |  | **r (p-value)** |
| **Copy of complex geometric figure (PG)** | -0.30 (0.26) | **Copy of complex geometric figure (PG)** | -0.01 (0.96) |
| **Copy of complex geometric figure (PC)** | -0.28 (0.30) | **Copy of complex geometric figure (PC)** | -0.04 (0.86) |
| **Objective decision** | -0.08 (0.77) | **Objective decision** | -0.25 (0.35) |
| **Progressive Silhouettes** | -0.21 (0.41) | **Progressive Silhouettes** | -0.04 (0.89) |
| **Position** | 0.09 (0.74) | **Position** | -0.04 (0.88) |
| **CADL-2** | 0.24 (0.36) | **CADL-2** | 0.07 (0.81) |
| **DAFS** | -0.06 (0.82) | **DAFS** | 0.11 (0.70) |

Abbreviations:

PC: correct score

PG: raw score

CADL2: Communication Activities of the Daily Living

DAFS: Direct Assessment of Functional Status
